# Long non-coding RNAs are involved in alternative splicing and promote cancer progression

**DOI:** 10.1038/s41416-021-01600-w

**Published:** 2021-11-08

**Authors:** Jiawei Ouyang, Yu Zhong, Yijie Zhang, Liting Yang, Pan Wu, Xiangchan Hou, Fang Xiong, Xiayu Li, Shanshan Zhang, Zhaojian Gong, Yi He, Yanyan Tang, Wenling Zhang, Bo Xiang, Ming Zhou, Jian Ma, Yong Li, Guiyuan Li, Zhaoyang Zeng, Can Guo, Wei Xiong

**Affiliations:** 1 NHC Key Laboratory of Carcinogenesis and Hunan Key Laboratory of Cancer Metabolism, Hunan Cancer Hospital and the Affiliated Cancer Hospital of Xiangya School of Medicine, Central South University, Changsha, Hunan China; 2 Key Laboratory of Carcinogenesis and Cancer Invasion of the Chinese Ministry of Education, Cancer Research Institute, Central South University, Changsha, Hunan China; 3 Hunan Key Laboratory of Nonresolving Inflammation and Cancer, Disease Genome Research Center, the Third Xiangya Hospital, Central South University, Changsha, Hunan China; 4 Department of Stomatology, Xiangya Hospital, Central South University, Changsha, Hunan China; 5 Department of Oral and Maxillofacial Surgery, The Second Xiangya Hospital, Central South University, Changsha, Hunan China; 6 Department of Medicine, Comprehensive Cancer Center Baylor College of Medicine, Houston, TX USA

**Keywords:** Non-coding RNAs, Cancer genetics

## Abstract

Alternative splicing (AS) is a key process in which precursor RNAs produce different mature RNAs, and the disorder of AS is a key factor in promoting cancer development. Compared with coding RNA, studies on the functions of long non-coding RNAs (lncRNAs) are far from enough. In fact, lncRNA is an important participant and regulator in the process of AS. On the one hand, lncRNAs regulate cancer progression as AS products of precursor messenger RNA (mRNA), but on the other hand, precursor lncRNA generates cancer-related abnormal splicing variants through AS. In addition, lncRNAs directly or indirectly regulate the AS events of downstream target genes, thus affecting the occurrence and development of cancer. Here, we reviewed how lncRNAs regulate AS and influence oncogenesis in different ways.

## Background

Alternative splicing (AS) refers to a process in which precursor RNAs (pre-RNAs) produce different splicing variants through different splicing patterns, mainly including exon skipping, intron inclusion, mutually exclusive exons and alternative 5′ and 3′ splice sites [1, 2]. However, more than 90% of human genes undergo AS [3], which is not only an important mechanism for regulating gene expression and generating proteome diversity, but is also a key factor leading to the large differences in the number of eukaryotic proteins. Specifically, different coding regions of a gene can be spliced in different ways, resulting in multiple transcription states of the gene, and the final protein product may have different or mutually antagonistic functional and structural characteristics. Differences in expression at the transcriptional and protein levels result in different phenotypes and changes in biological processes that may play important roles in development, differentiation and cause various diseases, including cancer [4, 5]. However, abnormal AS involved in the abnormal regulation of certain genes during tumorigenesis is far from sufficiently elucidated.

The main factor that causes the AS of pre-RNAs is the co-regulation of *cis*-elements and *trans*-acting factors; the *cis*-elements are exon splice enhancer, exon splice silencer, intron splice enhancer and intron splice silencer. Serine- and arginine-rich splicing factors (SR proteins) and heterogeneous nuclear ribonucleoproteins (hnRNPs) are two famous families of *trans*-acting factors [6, 7]. In addition, transcription, m6A modification and chromosome structure can affect AS [8, 9]. Thus, the occurrence of abnormal AS is also closely related to the mutation or disorder of these splicing-related elements, causing the occurrence of cancer. However, with further research on the function of non-coding RNAs, studies have found that non-coding RNAs have more unique and interesting functions in the process of AS [10], especially long non-coding RNAs (lncRNAs).

LncRNA is a type of RNA molecule with a transcript longer than 200 nucleotides. A unique function of lncRNAs is that they interact with various molecules and can form a variety of RNA–RNA, RNA–DNA, or RNA–protein complexes through specific RNA functional domains, leading to lncRNA functional diversity [11–14]. For this reason, lncRNAs, as important regulatory molecules, play a key biological function in the occurrence and development of cancer through different pathways [15–23]. Previous studies have shown that lncRNAs can be used as products of AS to play a biological function in promoting or suppressing cancer. Furthermore, lncRNAs may also participate in the abnormal AS process of certain cancer-related genes through the regulation of precursor messenger RNA (pre-mRNA), encoding small peptides and association with splicing factors and so on.

This review aimed to elucidate the role of lncRNAs involved in AS and their influence on tumorigenesis in order to identify new pathways for the study of cancer pathological mechanisms and clinical treatment.

### A product of AS of pre-mRNA: lncRNA

RNA splicing depends on the mutual cooperation of U1, U2, U4, U5 and U6 small nuclear ribonucleoproteins, and the whole process is completed by two transesterification reactions [2]. The selection of splice sites in pre-RNA induced by multiple splicing factors is the main reason for the occurrence of AS. However, the AS of human genes is common and extensive, and the birth of lncRNA is also inseparable from such a process. It may be generated as a splicing product, especially in the process of tumour development, where the abnormally expressed lncRNA directly affects its downstream pathways and plays a specific biological function [24–28].

**Fig. 1:**
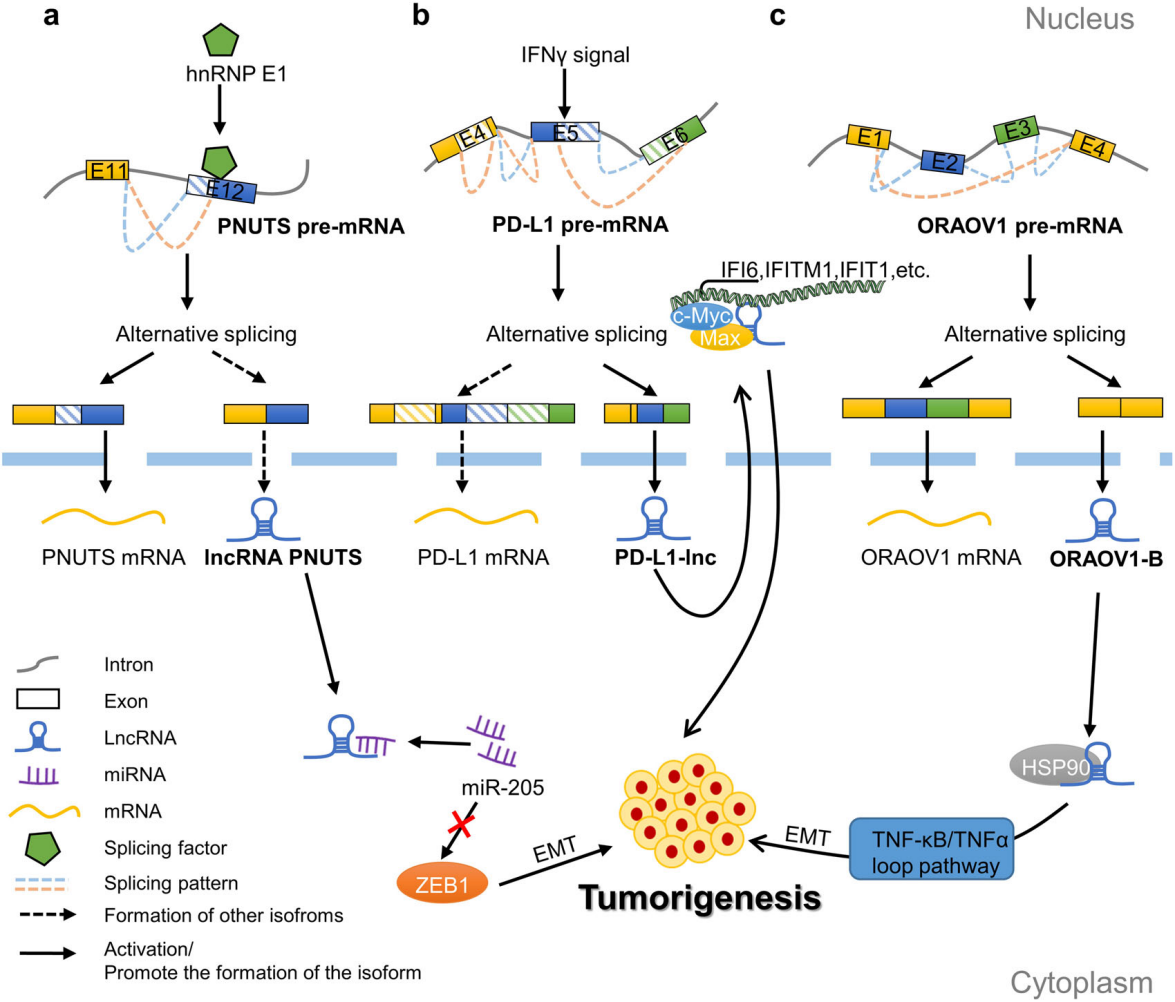
A product of AS of pre-mRNA: lncRNA. **a** Splicing factor hnRNPE1 promotes proximal 5′ splice‐site selection in exon 12, thus inducing the production of PNUTS mRNA. The absence of hnRNPE1 facilitates the generation of the lncRNA-PNUTS isoform, which promotes tumour development by competitively binding with miR-205 to upregulate ZEB1. **b** IFNγ promotes the generation of the PD-L1-lnc isoform and PD-L1-lnc activates the transcriptional activity of c-myc through the formation of a heterodimer with Max to promote the development of tumours. **c** In oral squamous cell carcinoma, exon 2 and 3 skipping of ORAOV1 pre-mRNA leads to the formation of the lncRNA ORAOV1-B isoform, which activates the TNF-κB/TNFα loop pathway by binding with HSP90.

Previous studies have shown that lncRNA-PNUTS splicing isoforms are involved in epithelial-to-mesenchymal transition (EMT)-related tumour progression. Moreover, the splicing inhibitor hnRNPE1 can bind to the PNUTS pre-mRNA exon 12 splicing site to regulate the AS of PNUTS. When hnRNPE1 binds to this site, PNUTS pre-mRNA undergoes normal splicing to produce PNUTS mRNA, whereas, when hnRNPE1 dissociates from this site, PNUTS pre-mRNA produces the non-coding isoform lncRNA-PNUTS. Functionally, lncRNA-PNUTS can act as a sponge to compete for miR-205, inhibit the binding of miR-205 to the target gene *ZEB1* and upregulate ZEB1, thereby inhibiting the expression of the epithelial marker E-cadherin and inducing EMT to promote tumorigenesis [29] (Fig. 1a). Similarly, researchers found that PD-L1 produces PD-L1-lnc through AS, which is composed of parts of exons 4, 5 and 6. Like PD-L1 mRNA, interferon-γ (IFNγ) can also upregulate PD-L1-lnc. Meanwhile, PD-L1-lnc can combine with chaperone protein Max to form a heterodimer to promote the nuclear distribution and transcriptional activity of c-Myc, thus enhancing the progression of cancer cells [30] (Fig. 1b). The lncRNA ORAOV1-B is a newly discovered AS variant of the *ORAOV1* gene, which activates the tumour necrosis factor-κB (TNF-κB)/TNFα loop by interacting with heat-shock protein 90 (HSP90), and then induces the EMT mechanism to promote the invasion and metastasis of oral squamous cell carcinoma [31] (Fig. 1c). These studies show that lncRNAs may play the role of cancer-related splicing products of pre-mRNA and regulate tumorigenesis.

### AS of pre-lncRNA produces generate different lncRNAs

**Fig. 2:**
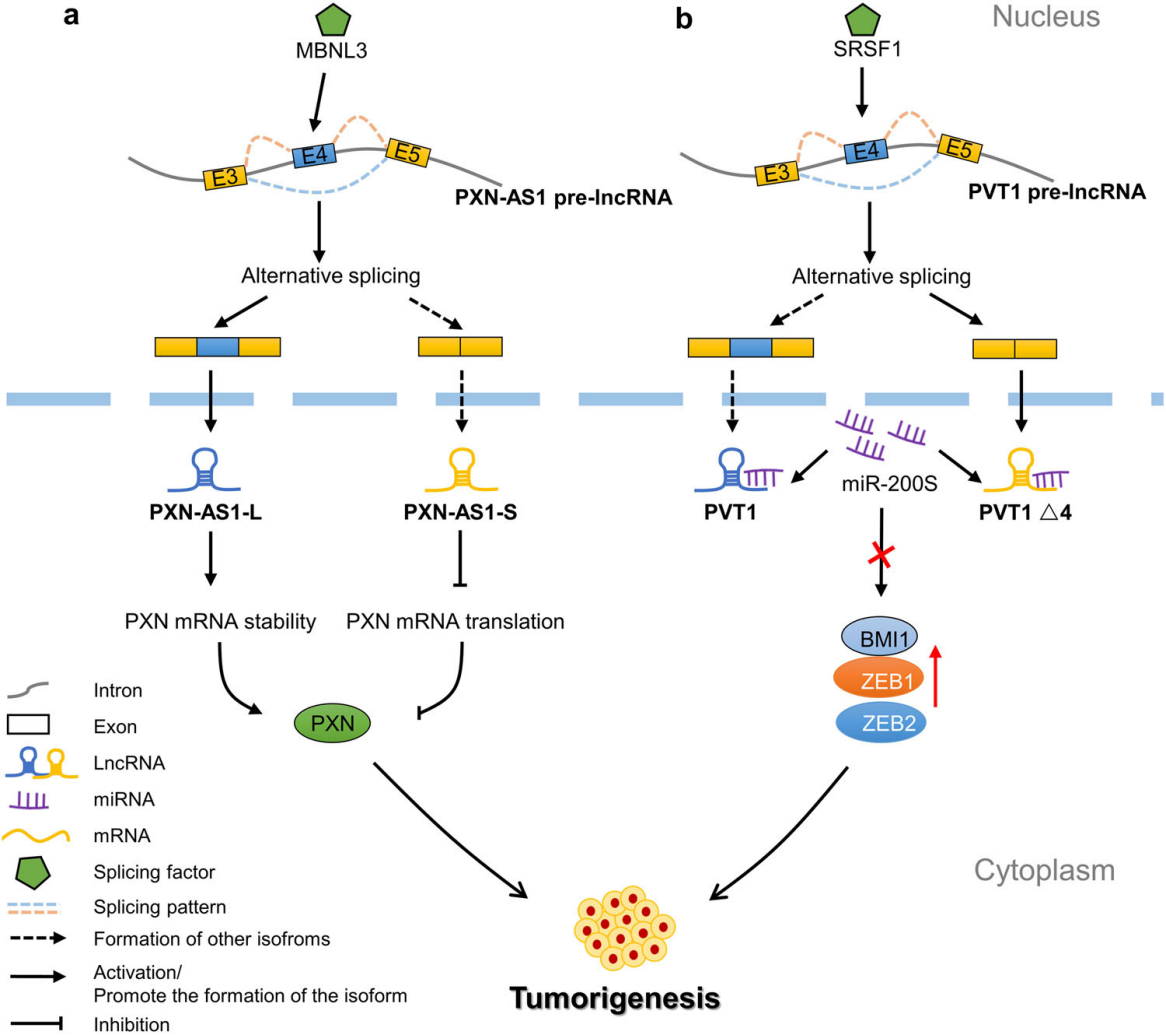
AS of pre-lncRNA produces generate different lncRNAs. **a** Through AS, PXN-AS1 pre-mRNA generates PXN-AS1-L and PXN-AS1-S, two lncRNA isoforms. Splicing factor MBNL3 promotes the inclusion of exon 4, thus upregulating PXN-AS1-L, promoting the stability of PXN mRNA and promoting the occurrence and development of tumours. On the contrary, PXN-AS1-S inhibits the translation of PXN mRNA, thereby inhibiting tumorigenesis. **b** PVT1 pre-lncRNAs generate PVT1Δ4 and PVT1 isoforms through AS. Splicing factor SRSF1 promotes exon 4 skipping, which is conducive to the generation of the PVT1 isoform. Both lncRNA variants can competitively bind to miR-200s to promote the upregulation of BMI1, ZEB1 and ZEB2, thereby inducing tumorigenesis.

LncRNA genes can also undergo AS and generate different lncRNA isoforms [32, 33]. However, the potential biological functions of most lncRNA isoforms have not been discovered, and more studies tend to focus on the variants with relatively high expression abundance. In fact, the functional mechanisms of lncRNA isoforms may be quite different [34]. It is proposed that splicing events at the lncRNA-associated enhancer RNA producing centre sites are associated with enhancer activity [35], but the findings of studies of the AS process of lncRNAs remain vague. MBNL3, a skeleton splicing factor, promotes tumorigenesis and indicates a poor prognosis for patients with hepatocellular carcinoma; it induces lncRNA-PXN-AS1 exon 4 inclusion by binding to lncRNA-PXN-AS1 intron 4 to promote liver cancer. The transcript of lncRNA-PXN-AS1 lacking exon 4 can bind to the coding sequence of PXN mRNA and lead to the dissociation of translation elongation factors from PXN mRNA, thereby inhibiting the translation of PXN mRNA and inhibiting liver cancer. In addition, the transcript containing exon 4 preferentially binds to the 3′-untranslated regions (UTR) of PXN mRNA and protects the PXN mRNA from degradation induced by the microRNA-24–AGO2 complex, thereby increasing PXN expression and promoting liver cancer [36] (Fig. 2a).

LncRNA PVT1 combines with miR-200S to upregulate the expression of ZEB1, ZEB2 and BMI1 and promotes tumour growth in clear-cell renal cell carcinoma (ccRCC). Interestingly, PVT1 lacking exon 4 (PVT1ΔE4) is a new splice isoform that has a high expression level in ccRCC. Splicing factor SRSF1 promoted the skipping of exon 4 of PVT1 and induced the expression of splicing variant PVT1ΔE4. It can also competitively bind with miR-200s to regulate the expression of ZEB1, ZEB2 and BMI1 as full-length transcripts, and the specific mechanism needs to be further studied [37] (Fig. 2b). In summary, these studies suggest that studying the differences in AS isoforms of lncRNAs is helpful to further understand the biological function of lncRNAs in the process of cancer development.

### LncRNAs directly participate in the regulation of AS

LncRNAs can be directly involved in the AS of target genes by means of their ability to form RNA–DNA duplexes [38] or RNA–RNA duplexes [39]. It is noteworthy that many of the lncRNAs that rely on complementary sequences to regulate AS are natural antisense transcripts.

#### LncRNAs regulate AS by establishing a splicing-specific chromatin tag

Whole-genome sequencing data revealed different chromatin landscapes on exon and intron sequences, confirming the role of chromatin as a splicing regulator. It has a unique splicing pattern by affecting the elongation of Pol II and specifically recruiting RNA-binding proteins that interact with chromatin-binding proteins [40]. The key issue in the regulation of chromatin-mediated splicing is the establishment and maintenance of regulatory chromatin domains. As partners of the chromatin modification complex and chromatin remodelling factor, lncRNAs play an important role in regulating the chromatin structure in tumours [41], which is highly likely to be a means for lncRNAs to regulate AS in tumours. However, the relationship between lncRNA and chromatin-mediated AS remains unclear.

**Fig. 3:**
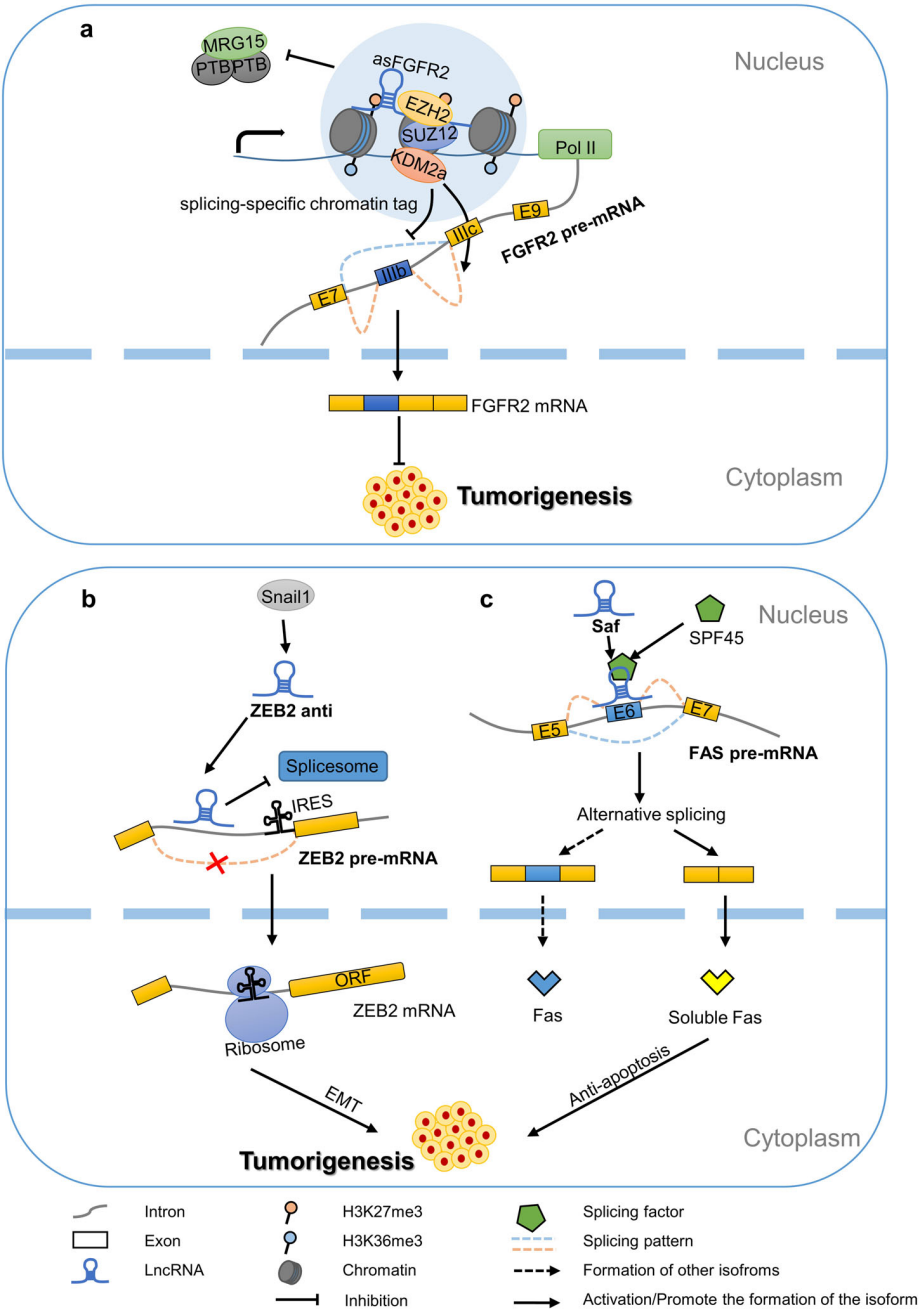
LncRNAs directly participate in the regulation of AS. **a** asFGFR2 recruits the EZH2 and SUZ12 of polycomb repressive complex 2 to its parent sites, triggering the recruitment of H3K36 demethylation enzyme KDM2a, thereby establishing a splicing chromatin tag that blocks the binding of chromatin adaptor complex MRG15-PTB to exon IIIb. This promotes the production of FGFR2 mRNA containing IIIb. **b** Snail1 promotes the formation of the RNA–RNA duplexes in introns of the 5′-UTR of parent RNA and prevents the removal of this intron during splicing. Then, the ribosome can be induced to recognise the internal ribosome entry site (IRES) in the intron and promote the protein expression of ZEB2. **c** Fas antisense transcription Saf binds to exon 6 of Fas pre-mRNA and recruits splicing factor SPF45 to facilitate the skipping of exon 6 and promote the generation of the sFas isoform, thereby inhibiting cell apoptosis and leading to tumour growth.

A previous study revealed that an evolutionarily conserved nuclear antisense lncRNA-asFGFR2 generated from the human FGFR2 locus promotes FGFR2-specific AS. asFGFR2 recruits the EZH2 and SUZ12 of polycomb repressive complex 2 and histone demethylase KDM2a to the FGFR2 locus, forming a splicing-specific chromatin tag to generate a chromatin environment; it blocks the binding of the chromatin adaptor complex MRG15-PTB to exon IIIb and eventually induces the inclusion of FGFR2 exon IIIb. It has been proposed that asFGFR2 is directly involved in AS by recruiting chromatin modifiers to the site through sequence complementarity and the formation of RNA–DNA double strands with the parent DNA [42]. The FGFR2 IIIb isoform plays an antitumor role in hepatocellular carcinoma, which provides a new approach for the study and treatment of this highly aggressive tumour [43] (Fig. 3a).

#### LncRNAs interact with target pre-mRNAs to regulate AS

The formation of RNA–RNA duplexes is a major function of lncRNAs, which is also performed by lncRNAs in AS. Interestingly, many antisense lncRNAs will form duplexes with their sense pre-mRNA and participate in AS.

One of the first discovered pre-mRNA directly regulated by its antisense lncRNA for AS is ZEB2. ZEB2 pre-mRNA has a long 5′-UTR, and effective translation requires retention of the 5′-UTR containing the intron of the internal ribosome entry site. However, the retention of introns depends on the expression of the antisense transcript (ZEB2-antisense RNA, ZEB2-anti) complementary to the 5′ splice site of the intron. In epithelial cells, ZEB2-anti is absent, and the exposure of the ZEB2 5′-UTR splicing site causes the splicing of the introns that regulate the translation of ZEB2. In mesenchymal cells, the mesenchymal marker Snail1 promotes the expression of ZEB2-anti, which combines with the ZEB2 5′-UTR intron to cover the splice donor that regulates the intron, inhibiting splicing and inducing translation to promote the occurrence of EMT [44] (Fig. 3b).

A major apoptosis pathway is triggered by the Fas receptor-Fas ligand (FasL) interaction. A lncRNA located in the nucleus, Fas antisense or Saf, interacts with the Fas pre-mRNA and splicing factor 45 to promote the cleavage of exon 6 [45]. The product is soluble Fas that can protect cells from Fas-FasL-induced apoptosis by binding to FasL (Fig. 3c).

In addition, BC200 has a carcinogenic effect in breast cancer. A study has found that BC200 contains a 17-nucleotide sequence complementary to Bcl-x pre-mRNA, which can promote its binding to Bcl-x pre-mRNA and the recruitment of the splicing factor hnRNPA2/B1. The hnRNPA2/B1 interferes with the binding of Bcl-x pre-mRNA and Bcl-xs promotion factor Sam68, resulting in the blockade of Bcl-xs expression, thereby promoting tumour occurrence [46]. Therefore, lncRNAs can interact with pre-mRNA and directly participate in the regulation of AS in cancers.

### LncRNAs are indirectly involved in the regulation of AS

#### LncRNAs regulate AS through the encoded peptide

Although lncRNAs belong to a class of RNA molecules that have no or weak coding ability, with the development of bioinformatics and high-volume measurement, small open-reading frames in lncRNAs have been gradually discovered, which can play a biological function by encoding small peptides. Many studies have shown that small peptides encoded by lncRNAs also play an important role in the development of cancer [47–51].

**Fig. 4:**
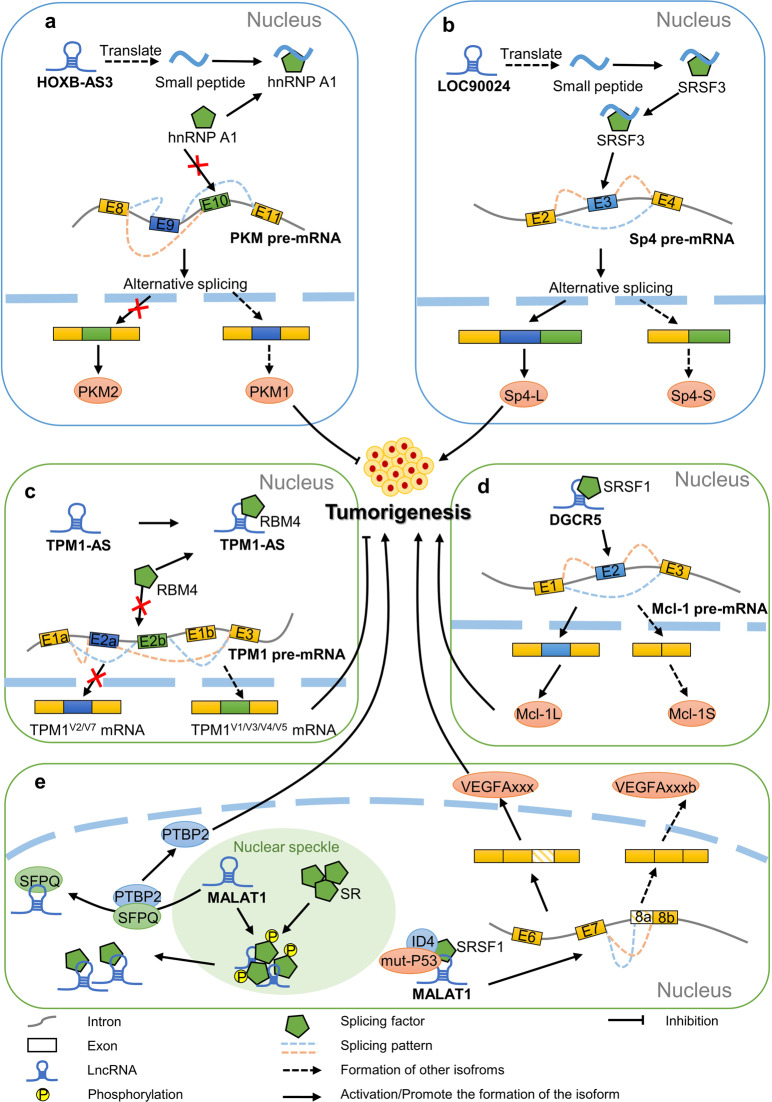
LncRNAs are indirectly involved in the regulation of AS. **a** PKM pre-mRNA can produce both PKM1 and PKM2 isoforms. HOXB-AS3 encodes a small peptide that competitively binds to the splicing factor hnRNPA1, thereby promoting the formation of PKM1 and ultimately inhibiting tumour growth. **b** Sp4 pre-mRNA can produce both long variants (Sp4-L) and short variants (Sp4-S) by AS. By interacting with SRSF3, the small peptide encoded by LOC90024 promotes the inclusion of exon 3 of Sp4 pre-mRNA and the generation of Sp4-L, which is conducive to tumour development. **c** TPM1-AS prevents the splicing effect of RBM4 on TPM1 by binding with the splicing factor RBM4, induces the generation of the TPM1 V1/V3/V4/V5 variant and inhibits the occurrence and development of tumour cells. **d** Mcl-1 pre-mRNA generates Mcl-1S (pro-apoptotic) and Mcl-1L (anti-apoptotic) by AS. The interaction between DGCR5 and SRSF1 promotes the retention of exon 2 and the generation of Mcl-1L. **e** MALAT1 hijacks SFPQ in the SFPQ/PTBP2 splicing factor complex, thereby causing the release of PTBP2 to promote tumour growth. Meanwhile, MALAT1 can control the transport and distribution of SR family proteins at transcription sites and among nuclear spots by affecting the phosphorylation of SR family proteins. MALAT1 forms a splicing complex with ID4, mut-P53 and SRSF1 to promote the distal 5′ splice‐site selection of exon 8, promoting the generation of angiogenic isoform VEGFA_xxx_ (the subscript ××× indicates the number of amino acids in different isoforms).

A previous study identified a small peptide encoded by the lncRNA HOXB-AS3, which was named 53-amino acid (aa) peptide, and showed that patients with colon cancer (CRC) with low levels of 53-aa peptide have a poor prognosis. The 53-aa peptide competitively binds to the arginine residue in the RGG motif of the splicing factor hnRNPA1 and blocks the binding of hnRNPA1 to the PKM pre-mRNA, thereby inhibiting the cleavage of PKM exon 9, resulting in the reduced synthesis of PKM2, an isoenzyme of glycolytic pyruvate kinase, thereby inhibiting the reprogramming of glucose metabolism and ultimately inhibiting the growth of CRC [52] (Fig. 4a).

In addition, lncRNA LOC90024-encoded small peptide SRSP can bind splicing factor SRSF3 to induce the inclusion of transcription factor Sp4 exon 3, promote the generation of Sp4-L while inhibiting Sp4-S and promote tumorigenesis of CRC [53] (Fig. 4b).

#### LncRNAs regulate AS by interacting with splicing factors

Splicing factors play a central role in the regulation of AS by interacting with the sequence elements of pre-RNA, while lncRNAs often play the role of cooperating with or hijacking splicing factors to regulate AS.

A study found TPM1-AS, an uncharacterised lncRNA, which was reverse transcribed from the second intron of TPM1. TPM1-AS is located at the nucleus and interacts with the splicing factor RBM4 in human oesophageal cancer cells. The interaction between TPM1-AS and RBM4 hinders the binding of RBM4 to TPM1 pre-mRNA and inhibits the inclusion of endogenous exon 2a of TPM1, resulting in the specific downregulation of TPM1 variants V2 and V7 in human oesophageal cancer cells, and producing the TPM1 mRNA variants V1, V3, V4 and V5, thereby inhibiting the migration and mitosis of human oesophageal cancer cells [54] (Fig. 4c).

In addition, lncRNA DGCR5 expression is upregulated in oesophageal squamous cell carcinoma (ESCC) and is associated with a poor prognosis. Researches have shown that DGCR5 interacts with SRSF1 and regulates the AS event of Mcl-1, which is conducive to the generation of the anti-apoptotic isoform Mcl-1L, thus promoting tumorigenesis [55] (Fig. 4d).

LncRNA MALAT1, as one of the classical lncRNAs associated with tumours, is highly expressed in many cancer types. Studies have shown that MALAT1 located in nuclear speckles can interact with a series of SR proteins such as SRSF1, SRSF2, SRSF3 and SRSF5 to varying degrees, and affect the transport and distribution of SR protein at transcription sites and between nuclear speckles by regulating the ratio of phosphorylation/dephosphorylation of SR proteins to regulate the AS events of pre-RNAs [56]. Previous studies have shown that MALAT1 can upregulate SRSF1-mediated series of cancer-promoting splicing events [57]. Another study showed that mutant p53 and ID4 proteins can form complexes with MALAT1 and SRSF1, regulate the AS of VEGFA pre-mRNA, inhibit the synthesis of the anti-angiogenic VEGFA isoform and promote angiogenic formation [58]. Not only does MALAT1 cooperate with the splicing factor but it also hijacks SFPQ from the splicing factor complex SFPQ/PTBP2 [59] (Fig. 4e).

Another study has shown that lincRNA-uc002yug.2 is generally overexpressed in ESCC. LincRNA-uc002yug.2 promotes the binding of SRSF1, MBNL1 and other splicing factors to the RUNX1 pre-mRNA, thereby upregulating the expression of RUNX1a and downregulating the expression of RUNX1 to reduce the expression level of the gene *CEBPα* and promote cell proliferation and tumorigenesis [60]. Meanwhile, in colorectal cancer, LINC01133 can interact with SRSF6 to inhibit the occurrence of EMT. Whether this lncRNA acts as a repressor of SRSF6 and, thus, affects the AS process of SRSF6-mediated EMT-related molecules remains to be studied [61]. In addition, linc01232 interacts with the splicing factor hnRNPA2/B1 and stabilises hnRNPA2/B1 by inhibiting hnRNPA2/B1 ubiquitination and degradation to promote the expression of its splicing target A-Raf full-length, thereby promoting pancreatic cancer [62]. These studies have revealed a new mechanism in which lncRNA regulates the splicing of target genes by interacting with splicing factors, thereby exerting a biological function of promoting or suppressing cancer.

### Bioinformatics tools for predicting lncRNAs and AS

Only a few studies have confirmed the close relationship between lncRNAs and AS. It is necessary to predict the AS events mediated by lncRNAs and the abundance of each transcript through bioinformatics methods. Most of the algorithms are based on the application of RNA-sequencing (RNA-seq) data sources and mathematical models, which will allow a more systematic and comprehensive understanding of the AS events of lncRNAs or the target genes regulated by them in cancers [63–65]. Deng et al. developed a cancer-related AS database of lncRNAs named lncAS2Cancer, which collected the RNA-seq data of more than 30 tumour types. The latest splicing algorithms (rMATS, MAJIQ, SEASTAR, Dapars, SUPPA2 and Brie) were used to obtain eight AS patterns of lncRNAs. Therefore, this tool is beneficial for predicting potential sequence functions, regulatory factors and biomarkers [66]. Meanwhile, in recent years, the RNA-seq data of liver cancer tissues and normal liver tissues from TCGA and GTEX-RNA were used to identify non-coding genes and AS isoforms with different expressions, and the physical interaction information network between lncRNAs and target genes was integrated through data mining. Finally, the RWMG model algorithm was used to analyse and predict the lncRNAs related to AS. This is the first time that a large-scale complex network has been integrated to predict the lncRNAs that regulate AS events in cancers, which is of great significance for discovering potential tumour markers [67].

## Perspective

As the most critical switch in RNA processing, AS strictly controls the physiological order at the transcriptome level and microregulates all aspects of biological effects in cells. Abnormal changes in AS make genes produce splicing isoforms after transcription and encode abnormal proteins. Splicing isoforms are related to cancer-related cell migration, cell growth regulation, hormone responsiveness, cell death and changes in gene expression in chemotherapy responses and become tumour biomarkers and molecular targets [68, 69]. There is evidence that splicing mutations affecting oncogenes, tumour suppressor genes and other cancer-related genes occur during the initiation and development of cancer and there is a causal link. However, the mechanism leading to abnormal splicing during tumorigenesis remains unclear. Various studies have shown that genetic and somatic mutations in *cis*-acting elements, as well as variations in the composition, concentration, location and activity of *trans*-regulators affect the recognition and function of splice sites and promote the development of cancers [5, 70, 71].

LncRNAs account for a considerable proportion of the human transcriptome. Due to the long length and poorly conserved characteristics of lncRNAs, their structure and function are much more complex than that of coding genes, but their main function is to regulate gene expression [15]. Recent studies have shown that lncRNA plays an important role in normal life activities and the development of diseases. In various human cancers, many lncRNAs are involved in transcriptional activation regulation, epigenetic regulation and signal transduction to affect the proliferation, apoptosis, invasion and metastasis, DNA damage repair, metabolism and drug resistance of cancer cells [72–79]. However, in the past decade, studies on the mechanism of lncRNAs participating in the process of AS have gradually attracted attention. Similarly, lncRNAs, as splicing regulators, affect these characteristics of malignant tumour cells.

For example, hypoxia-induced lncRNA LUCAT1 can cause chemotherapy resistance in colorectal cancer. These chemotherapy drugs are all DNA damage inducers, while lncRNA LUCAT1 causes AS of a series of DNA damage-related genes by binding splicing factor PTBP1 [80]. In addition, Ai-lncRNA EGOT can recruit hnRNPH1 to enhance the AS of pre-ITPR1 and form pre-ITPR1/EGOT double-stranded RNA to upregulate the expression of ITPR1, leading to the sensitivity of breast cancer cells to paclitaxel toxicity [81]. These findings suggest that it is very important to understand the mechanism of lncRNA-associated AS to solve the problem of drug resistance. Meanwhile, the AS events in the metabolic pathway regulated by lncRNAs are worthy of our attention. A study has found that lncRNA CCAT2 is involved in the AS process of glutaminase. Interestingly, CCAT2 is a lncRNA containing the single-nucleotide polymorphism (SNP) rs6983267. It reported that the allele of this SNP locus leads to different incidences of colorectal cancer. The authors found that different alleles of CCAT2 have different affinities for the subunit cleavage factor I 25 (CFlm25) and CFlm68 of CFlm, so that changes in the interaction between CCAT2, CFlm and glutaminase pre-mRNA result in different splicing results [82]. This ultimately leads to the metabolic reprogramming of tumour cells. Of course, lncRNAs are not only supporters of cancer-promoting splicing isoforms. LINC01348 interacts with splicing factor SF3B3 to promote the skipping of exon 14 of EZH2 pre-mRNA. Eventually, the LINC01348/SF3B3/EZH2/JNK/c-Jun/Snail pathway inhibits hepatocellular carcinoma progression [83]. LINC01133 has also been reported to interact with SRSF6 to inhibit EMT and metastasis in colorectal cells [61]. These studies suggest that lncRNA is extensively involved in the regulation of AS events in the tumorigenesis mechanism. In addition to cancer, lncRNAs also influence AS events in other diseases. The lncRNA GOMAFU can change the AS events of schizophrenia-related genes *DISC1* and *ERBB4*, and GOMAFU can interact with splicing factors QKI and SRSF1, which may be the mechanism of GOMAFU participating in the AS process [84]. The lncRNA Ctcflos is an important regulator of PRDM16, a key Brite fat-forming factor, which can regulate the splicing variant’s abundance of PRDM16, and then microregulate its function to regulate the production of Brite fat, which has research value in the treatment of obesity diseases and related comorbidities [85]. Therefore, the mechanism of lncRNA-associated AS in other diseases is worth in-depth study.

Understanding the potential relationship between lncRNAs and AS may find new treatment options for cancer treatment. Because of its selective anti-proliferative effect on cancer cells, the cold atmospheric plasma (CAP) is considered a potential alternative or complementary tool for cancer treatment and has been widely used in medicine in recent years. At present, studies have shown that CAP can kill a variety of tumours, but its tumour suppressor mechanism remains unclear. Studies have reported that CAP treatment of breast cancer cell line MCF-7 with different treatment conditions (600 s, 10 × 30 s) induces the opposite expression of ZNRD1 and its antisense lncRNA ZNRD1-AS1. When the CAP treatment condition is 600 s, the expression of ZNRD1 in MCF-7 cells is upregulated, while the expression of ZNRD1-AS1 is downregulated; when the CAP treatment condition is 10 × 30 s, the expression of ZNRD1 in MCF-7 cells is downregulated, while the expression of ZNRD1-AS1 upregulated [86]. This study has not yet discovered the specific mechanism of action between ZNRD1 and ZNRD1-AS1. However, the base complementary pairing of antisense lncRNA and sense mRNA is closely related to the AS mechanism, which is also likely to be one of the means to help formulate CAP treatment plans.

In general, the “butterfly effect mechanism” of lncRNA microregulates the expression of splicing isoforms of genes and then changes their functional effects in the mechanism pathway, which is of great research significance and provides new ideas and strategies for cancer research and clinical treatment. However, the involvement of lncRNAs in AS to regulate tumorigenesis is a relatively new research field. Few studies have investigated AS-related lncRNAs, and there is a need to develop effective systems biology approaches to develop a cancer-related lncRNA and AS events comprehensive network diagram to predict more relevant splicing isoforms of lncRNA and discover new AS mechanisms to identify more potential biomarkers. In addition, one of the major challenges in current research is the lack of experimental animal models related to splicing isoforms, which is indispensable for linking basic research with clinical practice. Another challenge is the development of effective targeted drugs that target small molecules that regulate key cancer-related AS events, such as lncRNAs, and the development of splicing drugs will be a significant step towards personalised therapy in the future.

## Conclusion

In this review, we discussed the mechanism of how lncRNAs participate in the regulation of AS in different ways and how they are involved in the regulation of certain tumorigenesis processes (Table 1). Specifically, lncRNAs can either become a cancer-associated splicing isoform or regulate the generation of cancer splicing variants of target genes. This may help us to further understand the molecular mechanism of cancer occurrence and development and find a new therapeutic method for cancer treatment.

**Table 1:** List of mechanisms by which lncRNA participates in AS.

LncRNA name	Classification of AS	Mechanism of AS	Function	Tumour types	Reference
PNUTS	An AS product of pre-mRNA	An AS product of PUNTS pre-mRNA, the absence of hnRNPE1 promotes the production of this lncRNA	Competitively binds with miR-205 to upregulate ZEB1 to promote tumorigenesis	Lung adenocarcinoma, breast cancer, colorectal adenocarcinoma	[29]
PD-L1-lnc	An AS product of pre-mRNA	An AS product of PD-L1 pre-mRNA, IFNγ promotes the formation of PD-L1-lnc	Binds to chaperone Max to form a heterodimer to promote the nuclear distribution and transcriptional activity of c-Myc, thus promoting cancer	Lung cancer	[30]
ORAOV1-B	An AS product of pre-mRNA	An AS product of ORAOV1 pre-mRNA	Combines Hsp90 activation of TNF-κB/TNFα loop and predominately promotes tumorigenesis	Oral squamous cell carcinoma	[31]
PXN-AS1-S	An AS product of pre-lncRNA	An AS product of pre-lncPXN-AS1, splicing factor SRSF1 promotes the exon 4 skipping of PXN-AS1 and generates this isoform	Inhibits the translation of PXN, thus promoting tumorigenesis	Hepatocellular carcinoma	[36]
PVT1ΔE4	An AS product of pre-lncRNA	An AS product of pre-lncPVT1, splicing factor SRSF1 promotes the exon 4 skipping of PVT1 to produce this isoform	Competitively binds with miR-200s to regulate the expression of ZEB1, ZEB2 and BMI1 to promote tumorigenesis	Clear-cell renal cell carcinoma	[37]
CRNDE-g	An AS product of pre-lncRNA	An AS product of pre-lncCRNDE	Upregulated in a variety of tumour types	Colorectal cancer, glioma, acute myeloid leukaemia	[87]
LHFPL3-AS1-long	An AS product of pre-lncRNA	An AS product of pre-lncLHFPL3-AS1, splicing factor PTBP1 interacts with the exon 3 of pre-lncRNA, resulting in the formation of the isoform	Directly interacts with miR-181a-5p to inhibit the degradation of Bcl-2 mRNA, thereby inhibiting cell apoptosis	Melanoma	[88]
SOX2OT V4 /V7	An AS product of pre-lncRNA	An AS product of pre-lncSOX2OT	Upregulated in tumours	Non-small cell lung tumour	[89]
asFGFR2	Establishing a splicing-specific chromatin tag	Establishes a splice-specific chromatin tag that damages the chromatin adaptor complex MRG15-PTB binding to the gene, and induces the inclusion of FGFR2 exon IIIb	Promotes the generation of the antitumor FGFR2 IIIb isoform by regulating AS	Hepatocellular carcinoma	[42]
ZEB2-anti	Binding to the target pre-mRNA	Binds to ZEB2 pre-mRNA to inhibit intron splicing at the 5′-UTR to induce ZEB2 translation	Promotes ZEB2 expression and induces tumorigenesis	Breast cancer	[44]
UXT-AS1	Binding to the target pre-mRNA	Regulates the alternative splicing at the 5′ end of UXT pre-mRNA to generate the UXT1 isoform	Promotes tumorigenesis by regulating the AS of UXT	Colon cancer	[90]
Saf	Binding to the target pre-mRNA	Binds to the exon 6 of Fas pre-mRNA and recruits splicing factor SPF45 to cooperatively regulate AS and promote the generation of the sFas isoform	Promotes the generation of the sFas isoform by regulating AS to block the Fas-FasL-induced apoptotic pathway	Erythroleukemia, cervical carcinoma	[45]
BC200	Binding to the target pre-mRNA	Binds to Bcl-x pre-mRNA and recruits hnRNPA2/B1 to co-regulate the AS of Bcl-x and inhibit the formation of the Bcl-xs isoform	Induces tumorigenesis by regulating AS to inhibit the generation of the pro-apoptotic Bcl-xs isoform	Breast cancer	[46]
Ai-lncRNA EGOT	Binding to the target pre-mRNA	Promotes ITPR1 AS by recruiting hnRNPH1 by binding to ITPR1 pre-mRNA	Promotes the expression of ITPR1 by regulating AS and enhances the sensitivity of cells to paclitaxel	Breast cancer	[81]
PLANE	Binding to the target pre-mRNA	Cooperates with hnRNPM to regulate an alternative 5′ splice site within intron 45 selection of NCOR2 pre-mRNA to generate NCOR2-001/005-like mRNA variants	Promotes the development of tumours by regulating the AS process of NCOR2 pre-mRNA	Lung adenocarcinoma, breast cancer	[91]
HOXB-AS3	Encoding small peptides and mediating alternative splicing	The encoding peptide hijacks the splicing factor hnRNPA1 to induce exon 9 to include PKM pre-mRNA and promote PKM1 formation	Promotes the generation of the PKM1 isoform through AS and regulates the metabolic reprogramming of tumour cells	Colon cancer	[52]
LOC90024	Encoding small peptides and mediating alternative splicing	The small peptide SRSP binds to the splicing factor SRSF3 to induce the exon 3 skipping of Sp4 and promote the formation of the Sp4-L isoform	Promotes tumorigenesis and progression by inducing the formation of the Sp4-L isoform through AS	Colorectal cancer	[53]
LincRNA-uc002yug.2	Interacting with splicing factors	Cooperates with SRSF1 and MBNL1 to regulate the AS of RUNX1 pre-mRNA and promote the generation of RUNX1a	Induces the generation of the RUNX1a isoform by AS and leads to the decrease of CEBPα expression, which promotes tumorigenesis	Oesophageal squamous cell carcinoma	[60]
SNHG6	Interacting with splicing factors	SNHG6 can target PKM mRNA and induce hnRNPA1-specific splicing of PKM pre-mRNA	Leads to metabolic reprogramming of tumour cells by regulating AS	Colorectal cancer	[92]
TPM1-AS	Interacting with splicing factors	Hijacks RBM4 to prevent its binding to TPM1 pre-mRNA and inhibits exon 2A inclusion, inducing downregulation of the expression of V2 and V7 variants	Inhibits migration and mitotic formation of human oesophageal cancer cells by regulating the AS of TPM1 pre-mRNA	Oesophageal cancer	[54]
Linc01232	Interacting with splicing factors	Participates in the AS of A-Raf by stabilising HNRNPA2B1 and promoting the generation of the A-RAF-FL isoform	The Linc01232/hnRNPA2B1/A-Raf /MAPK axis promotes tumorigenesis	Pancreatic cancer	[62]
DGCR5	Interacting with splicing factors	Cooperates with SRSF1 to regulate the AS of Mcl-1 pre-mRNA and the inclusion of exon 2 to induce the production of Mcl-1L	Induces tumorigenesis by regulating AS to promote the generation of anti-apoptotic Mcl-1L	Squamous cell carcinoma of the oesophagus	[55]
CRNDE	Interacting with splicing factors	Promotes the generation of the PICALML isoform by stabilising SRSF6 involved in the alternative splicing of PICALM pre-mRNA and the inclusion of exon 14	Regulates the AS of PICALM pre-mRNA and is involved in autophagy-induced drug resistance in tumour cells	Gastric cancer	[93]
LUCAT1	Interacting with splicing factors	Cooperates with PTBP1 to regulate AS of APP, CD44, CLSTN1, MBNL1 and ZNF207	Regulates the AS of a series of genes related to DNA damage and induces drug resistance of tumour cells	Colorectal cancer	[80]
PCGEM1	Interacting with splicing factors	Interacts with splicing factors hnRNPA1 and U2AF65 to regulate the AS of androgen receptor (AR) pre-mRNA and the expression of AR3 variants	Regulates the expression of AR3 by interacting with splicing factors	Prostate cancer	[94]
PNCTR	Interacting with splicing factors	Hijacks PTBP1 to regulate the AS of CHEK pre-mRNA and the inclusion of exon 8, thereby promoting the generation of the CHEK-L isoform and the development of tumours	Promotes the expression of CHEK-L through the AS mechanism and induces tumorigenesis	Cervical cancer	[95]
LINC01348	Interacting with splicing factors	LINC01348 complex with splicing factor 3B subunit 3 (SF3B3) acts as a modulator of EZH2 pre-mRNA and promotes the skipping of exon 14 of target gene *EZH2* pre-mRNA	LINC01348/SF3B3/EZH2/JNK/c-Jun/Snail signalling pathways inhibit tumorigenesis	Hepatocellular carcinoma	[83]
MALAT1	Interacting with splicing factors	(1) Cooperates with SRSF1 to regulate the AS of BIM, BINI, TEAD1 and VEGFA pre-mRNA (2) Regulates the localisation and distribution of SR protein family in the nucleus (3) Hijacks the SFPQ in the splicing factor complex SFPQ/PTBP2 and releases the splicing factor PTBP2	Regulates splicing factors and indirectly affects the AS of cancer-related isoforms, thus promoting the development of tumours	Breast cancer, hepatocellular carcinoma, ovarian cancer	[57, 58, 96]

## Data Availability

Not applicable.
